# Asymmetrical bipolar nanosecond electric pulse widths modify bipolar cancellation

**DOI:** 10.1038/s41598-017-16142-6

**Published:** 2017-11-27

**Authors:** Chris M. Valdez, Ronald A. Barnes, Caleb C. Roth, Erick K. Moen, Graham A. Throckmorton, Bennett L. Ibey

**Affiliations:** 1 National Research Council Research Associateship Program, Air Force Research Laboratory, 4141 Petroleum Rd., JBSA Fort Sam Houston, Texas, 78234 USA; 2 Radio Frequency Bioeffects Branch, Bioeffects Division, Airman Systems Directorate, 711th Human Performance Wing, Air Force Research Laboratory, 4141 Petroleum Rd., JBSA Fort Sam Houston, Texas, 78234 USA; 3 Ming Hsieh Department of Electrical Engineering- Electrophysics, University of Southern California, 920 Bloom Walk, SSC, 502, Los Angeles, California USA; 4 Department of Biomedical Engineering, Vanderbilt University, 2301 Vanderbilt Place, Nashville, Tennessee, 37235 USA

## Abstract

A bipolar (BP) nanosecond electric pulse (nsEP) exposure generates reduced calcium influx compared to a unipolar (UP) nsEP. This attenuated physiological response from a BP nsEP exposure is termed “*bipolar cancellation*” (*BPC*). The predominant BP nsEP parameters that induce *BPC* consist of a positive polarity (↑) front pulse followed by the delivery of a negative polarity (↓) back pulse of equal voltage and width; thereby the duration is twice a UP nsEP exposure. We tested these *BPC* parameters, and discovered that a BP nsEP with symmetrical pulse widths is not required to generate *BPC*. For example, our data revealed the physiological response initiated by a ↑900 nsEP exposure can be *cancelled* by a second pulse that is a third of its duration.  However, we observed a complete loss of *BPC* from a ↑300 nsEP followed by a ↓900 nsEP exposure. Spatiotemporal analysis revealed these asymmetrical BP nsEP exposures generate distinct local YO-PRO®-1 uptake patterns across the plasma membrane. From these findings, we generated a conceptual model that suggests *BPC* is a phenomenon balanced by localized charging and discharging events across the membrane.

## Introduction

A nanosecond electric pulse (nsEP) is defined as a high intensity electric field (1.0–50 kV/cm) applied over a nanosecond time scale. Biological tissue exposed to a nsEP can exhibit altered physiology both immediately and days after exposure^1^. For example, a nsEP exposure can generate membrane perturbations that facilitate entry of small ions into the cell causing membrane depolarization, intracellular second messenger signaling, and apoptotic pathway activation^2–4^. These various cellular effects can be tuned by modulating nsEP intensity (duration, amplitude, and number of pulses) highlighting the mechanistic depth and breadth of its impact on biological systems. A prime example are responses generated from exposure to a unipolar (UP) or bipolar (BP) nsEP^5^. Cells exposed to a BP nsEP exhibit reduced calcium and propidium uptake as compared to the same total duration UP exposures, suggesting the BP nsEP generates less membrane perturbation^6^. Interestingly, this reduced membrane permeabilization was also detected when the pulse duration was doubl that of the UP nsEP^7^. Specifically, the Ca^2+^ influx initiated from a UP nsEP exposure was “*cancelled*” due to the delivery of the second pulse that was opposite in polarity and equivalent in pulse duration. Moreover, this effect, termed *bipolar cancellation* (*BPC*), was still observed when the amplitude of the second pulse was reduced to 35% of the front pulse^2^. Remarkably, increasing the interpulse interval between the two waveforms of the BP nsEP can partially resolve *BPC* of calcium uptake^7^.

Currently, the delivery of asymmetrical BP pulses is specific to microsecond pulse exposures; however, the data demonstrates that asymmetry across the individual BP pulse widths can generate lethal thresholds from irreversible electroporation that are similar to high frequency unipolar applications^8^. These observations extend the possibility that asymmetry across BP nsEP widths may modify our current understanding of *BPC*. To this end, we evaluated if the physiological response initiated by a BP nsEP that exhibits asymmetry among individual pulse widths would *cancel* the physiological effect initiated by the first pulse. We also measured local membrane perturbations from the anode and cathode side of the cell through second harmonic generation (SHG) imaging, and completed a spatiotemporal analysis of YO-PRO®-1 uptake, a marker of membrane integrity.

Our results demonstrated that *BPC* is modified when individual BP nsEP widths are asymmetrically tuned. Furthermore, our data revealed that *BPC* is maintained upon delivery of a second pulse that is one third of the front pulse duration; however, a second pulse exposure, opposite in polarity, that is three times the duration of the first results in a loss of *BPC*. Our spatiotemporal analysis confirmed that exposure from a BP nsEP with symmetrical pulse widths generates equivalent YO-PRO®-1 uptake on both sides of the cell; thereby increasing the uptake of small molecules into the cell more symmetrically, as previously described^9^. Furthermore, spatiotemporal analysis revealed that placement of the longer-duration portion of the asymmetric BP nsEP correlated to the side of the cell with peak YO-PRO®-1 fluorescence. To summarize our findings, we propose a working model that describes the spatial aspects of YO-PRO®-1 uptake generated from symmetrical and asymmetrical BP nsEP exposures.

## Results

### Symmetrical bipolar cancellation

**Figure 1:**
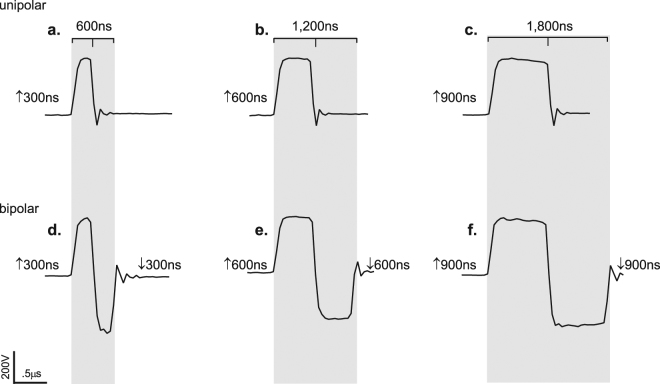
Oscilloscope traces of unipolar (UP) and symmetrical bipolar (BP) nanosecond pulses (nsEPs). (**a**–**c**) The UP nsEPs are of a single positive polarity (↑) pulse ranging from 300, 600 and 900 nanoseconds (ns). (**d**–**f**) The BP nsEPs consist of a ↑300, ↑600, or ↑900 ns front pulse, and are considered symmetrical because the front pulse is followed by a negative polarity pulse (↓) of the same duration, and peak-to-peak voltage.

**Figure 2:**
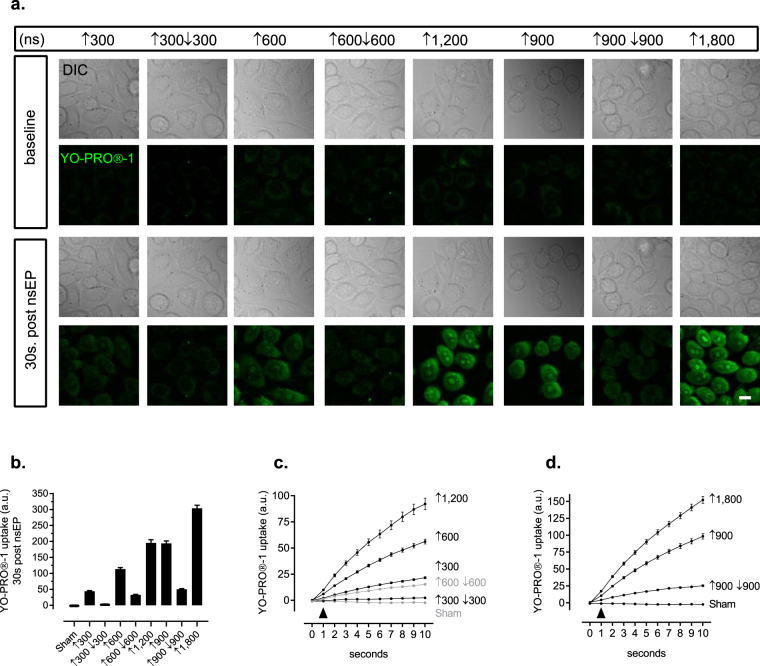
Symmetrical BP nsEPs that double the energized time of the front nsEP exhibit bipolar cancellation (BPC). (**a**) Representative differential interference contract (DIC) and confocal micrographs of Chinese Hamster Ovarian-K1 (CHO-K1) cells exposed to ↑300, ↑600, or ↑900 UP nsEPs, and symmetrical ↑300↓300, ↑600↓600, and ↑900↓900 BP nsEPs. The confocal micrographs represent YO-PRO®-1 fluorescence at baseline and 30 s post the nsEP exposure; scale bar 10 µm. (**b**) Quantitative assessment of YO-PRO®-1 uptake following exposure to UP and symmetrical BP nsEPs. In the plot, we evaluate YO-PRO®-1 uptake from UP nsEPs, and BP nsEP exposures that exhibit equivalent pulse durations (i.e. ↑300 ns vs. ↑300↓300 ns). As a control, we also measured YO-PRO®-1 uptake from UP nsEP exposures that have the same total energized time as the BP nsEPs (i.e. ↑1,200 ns vs. ↑600↓600 ns). Quantitative statistics- nsEP exposure, cells (N, cells): Sham (11,130), ↑300 (11,146), ↑600 (11,151), ↑900 (11,123), ↑1,200 (8,93), ↑1,800 (8,107), ↑300↓300 (11,129), ↑600↓600 (11,141), and ↑900↓900 (11,133). Statistical analysis of YO-PRO®-1 uptake 30 s post the nsEP exposure was determined with an unpaired two-tailed *t-test*, alpha = 0.05, p < 0.0001; (**c**,**d**) Temporal profile of YO-PRO®-1 uptake for 10 s following exposure to symmetrical BP nsEPs, and UP nsEPs. The zero point on the x-axis marks baseline YO-PRO®-1 fluorescence, and the black arrow head labels delivery of the nsEP. Nanosecond electric pulse exposures with similar temporal profiles are distinguished in gray to enhance contrast between the data sets.

To evaluate if *BPC* directly alters membrane permeability, we exposed CHO-K1 cells to ↑300 and ↑300↓300 nsEPs in the presence of YO-PRO®-1 (Fig. 1a,d). From live confocal microscopy, we collected 10 s of baseline images followed by 30 s post nsEP exposure (Fig. 2a). At 30 s post nsEP delivery, CHO-K1 cells exhibited a 91.2% decrease in YO-PRO®-1 uptake from the ↑300↓300 nsEP exposure compared to a ↑300 nsEP (Fig. 2b). Furthermore, we compared YO-PRO®-1 uptake generated from a ↑600 nsEP, which exhibits equivalent total energized time to the ↑300↓300  nsEP (Fig. 1a,d). Despite the comparable duration, we observed a 96.5% decrease in YO-PRO®-1 uptake from the ↑300↓300 nsEP exposure compared to the ↑600 nsEP (Fig. 2a,b).

Previous research demonstrated that pulse duration can differentially influence the extent of nsEP-induced membrane permeability in cells^1^. For this reason, we evaluated *BPC* from BP nsEP exposures with increasing symmetrical ↑600 and ↑900 ns pulse widths (Fig. 1b,c,e,f). From our confocal micrographs, we observed a difference in YO-PRO®-1 uptake between the ↑600 and ↑600↓600 nsEP exposures, respectively (Fig. 2a). Upon quantification, our data revealed that a ↑600↓600 nsEP generated a 71% decrease in YO-PRO®-1 uptake compared to a ↑600 nsEP, and an 83.2% decrease compared to a 1.2µs UP electric pulse exposure (Fig. 2b). Furthermore, our analysis demonstrated that a ↑900↓900 nsEP exposure generated a 74% decrease in YO-PRO®-1 uptake in comparison to ↑900 nsEP exposure (Fig. 2b). As a control, we compared YO-PRO®-1 uptake from a ↑900↓900 nsEP exposure to a 1.8µs UP electric pulse, and observed a 83.4% reduction in dye uptake from the BP nsEP exposure despite an equivalent total pulse duration (Fig. 2b).

Thus far, our investigation of *BPC* assessed YO-PRO®-1 uptake at 30 s post nsEP exposure. To evaluate if these reduced YO-PRO®-1 levels are the product of attenuated uptake over time, we measured YO-PRO®-1 uptake for the first 10 s following the nsEP exposure. At 2 s post nsEP delivery, we observed differences in YO-PRO®-1 uptake among the ↑300↓300 and ↑600↓600 compared to the ↑300 and ↑600 nsEP exposures, respectively (Fig. 2d). More so, we compared the temporal profile of YO-PRO®-1 uptake following a ↑900 and ↑900↓900 nsEP exposure. Similarly, we observed differences in YO-PRO®-1 uptake that began 2 s post nsEP delivery (Fig. 2d).

### Asymmetrical BP nsEP widths and total pulse duration alters the extent of bipolar cancellation

**Figure 3:**
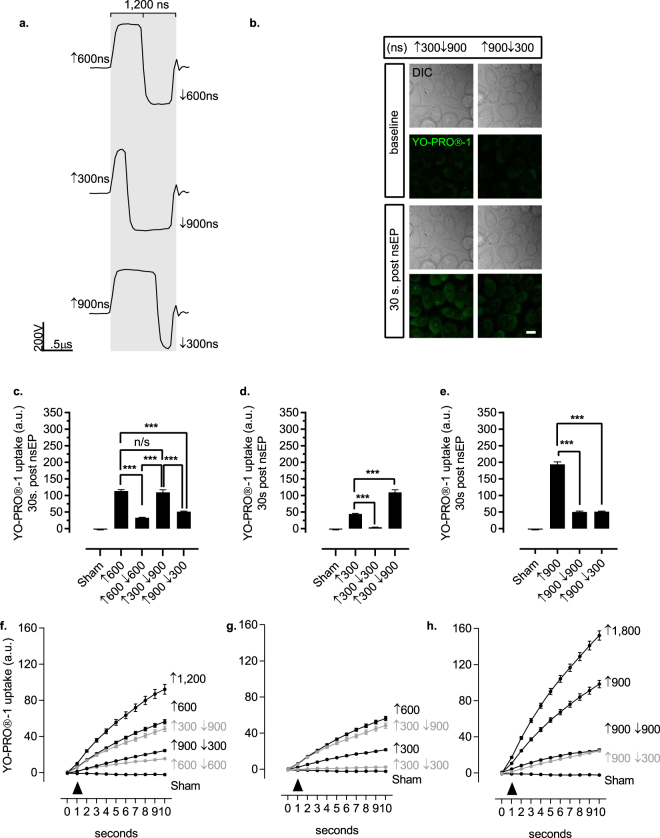
Altered total energized times in asymmetrical BP nsEPs effect *bipolar cancellation* *(BPC)*. (**a**) BP nsEP oscilloscope traces where the ↑600↓600 BP exhibits symmetrical and opposite polarity pulses resulting in double the energized time compared to a ↑600 nsEP. The following BP nsEPs also maintain a doubling of total energized time, but individual pulse durations are asymmetrically divided. (**b**) Representative DIC and confocal micrographs of CHO-K1 cells exposed to asymmetrical ↑300↓900 and ↑900↓300 nsEPs. YO-PRO®-1 uptake is represented at baseline then 30 s following the nsEP exposure; scale bar 10 µm. (**c**) YO-PRO®-1 uptake from a BP nsEP exposure composed of asymmetrically divided pulses that maintain a total energized time equivalent to a symmetrical ↑600↓600 BP nsEP. Quantitative statistics (nsEP exposures, cells): ↑300 ↓900 (10,74), and ↑900 ↓300 ns (11,150). Statistical analysis: unpaired two-tailed *t-test*, alpha = 0.05, ***p < 0.0001; (**d**) YO-PRO®-1 uptake upon a BP nsEP exposure where the second pulse has a three-fold increase in pulse duration compared to delivery of the first nsEP. Statistical analysis: unpaired two-tailed *t-test*, alpha = 0.05, ***p < 0.0001; (**e**) YO-PRO®-1 uptake upon exposure a BP nsEP where the second pulse has a three-fold decrease in pulse duration compared to the first opposite polarity pulse. Statistical analysis: unpaired two-tailed *t-test*, alpha = 0.05, ***p < 0.0001; (**f**) Temporal profile of YO-PRO®-1 uptake for 10 s following nsEP exposure (black arrow head). The ↑300↓900 and the ↑900↓300 nsEPs asymmetrically divide the front and back pulse durations, but maintain the total energized time found in the ↑600↓600 nsEP. (**g**) Temporal plot of YO-PRO®-1 uptake, 10 s post a BP nsEP exposure that asymmetrically divides the opposite polarity pulses where the second pulse has a three-fold increase in pulse duration compared to the first nsEP. (**h**) YO-PRO®-1 uptake for 10 s post a BP nsEP exposure that asymmetrically divides opposite polarity pulses where the second pulse has a three-fold decrease in pulse duration compared to the first nsEP.

To evaluate the physiological effect driven by asymmetrical BP nsEP widths, we constructed two BP nsEPs with different front and back pulse widths (i.e. ↑600 vs. ↑300↓900 and ↑900↓300 nsEPs) (Fig. 3a). We exposed CHO-K1 cells to the ↑300↓900 and ↑900↓300 nsEPs, and evaluated YO-PRO®-1 uptake compared to a ↑600 nsEP exposure (Fig. 3b). In comparison to the ↑600 nsEP exposure, CHO-K1 cells exhibit no significant change in YO-PRO®-1 uptake from a ↑300↓900 nsEP exposure, but a 55% reduction from a ↑900↓300 asymmetrical nsEP delivery (Fig. 3c). While these BP nsEPs consist of asymmetrical pulse widths they also exhibit a total pulse duration that is twice the ↑600 nsEP- a characteristic of the previously described *BPC* paradigm^7^. To test if a BP nsEP exposure will exhibit attenuated YO-PRO®-1 uptake only if the total pulse duration is twice the UP nsEP, we compared the biological effect generated from a ↑300↓900 versus a ↑300 nsEP exposure. We observed a 147.15% increase in YO-PRO®-1 uptake from a ↑300↓900 nsEP exposure compared to a ↑300 nsEP (Fig. 3d). We next evaluated a ↑900↓300 nsEP to a ↑900 nsEP exposure, and observed a 73.72% decrease in YO-PRO®-1 uptake from the ↑900↓300 nsEP compared to a ↑900 nsEP exposure, which percent change was similarly observed from a ↑900↓900 nsEP presentation (Figs 3b and 2d). We also evaluated the temporal profile of YO-PRO®-1 uptake when the BP nsEPs were asymmetrical in pulse width, but deviated from a doubling of total pulse duration. In response to a ↑300↓900 nsEP exposure, the YO-PRO®-1 uptake temporal profile resembled that of a ↑600 ns pulse (Fig. 3f,g). Furthermore, in comparison to the ↑900 ns pulse, the ↑900↓300 nsEP generated a temporal kinetic similar to the symmetrical ↑900↓900 nsEP; moreover, both BP nsEPs generated less YO-PRO®-1 uptake compared to a single 1.8µs UP exposure (Fig. 3h).

### Spatiotemporal dynamics of bipolar cancellation

**Figure 4:**
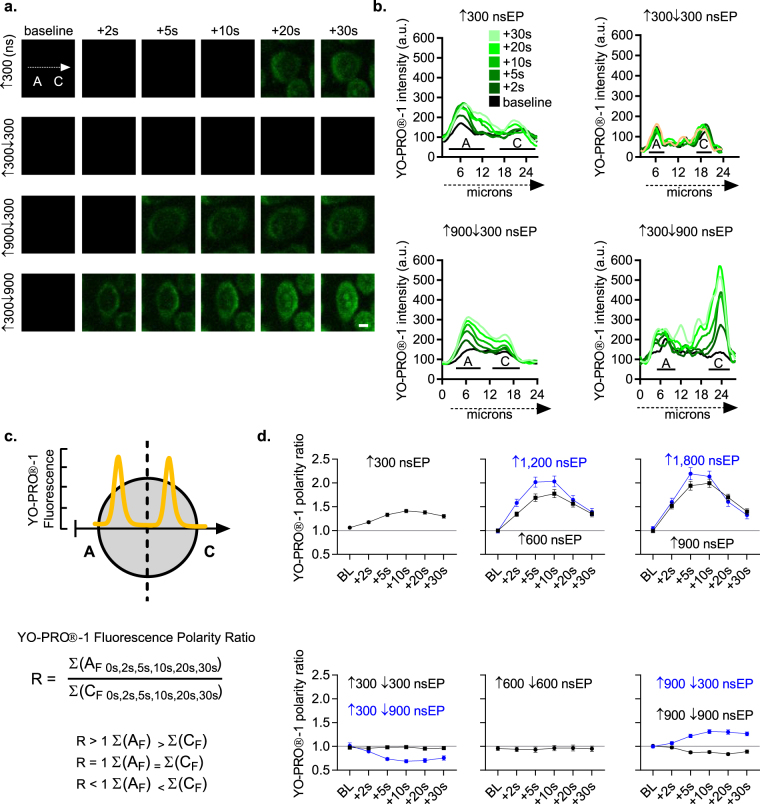
Spatiotemporal analysis of symmetrical and asymmetrical BP nsEPs. (**a**) Representative images of YO-PRO®-1 fluorescence taken at baseline and 2, 5, 10, 20, and 30 s following the nsEP exposure. The spatial distribution of YO-PRO®-1 fluorescence was captured with a line profile that began at the “*anode*” and ended on the “*cathode*” side of the cell; scale bar 5 µm. (**b**) The spatial and temporal distribution of YO-PRO®-1 fluorescent intensity over a single cell. The values are collected from a line profile (dotted arrow) that begins on the “*anode*” and ends on the “*cathode*” side of the cellular membrane. The black line marks YO-PRO®-1 fluorescence at baseline, and the warmer green lines identify YO-PRO®-1 fluorescence at 2, 5, 10, 20, and 30 s post nsEP exposure. (**c**) Schematic that represents our spatiotemporal assessment of a YO-PRO®-1 polarity ratio. The polarity ratio was calculated by dividing the sum of YO-PRO®-1 fluorescence from the “*anode*” and “*cathode*” sides of the cell to generate a YO-PRO®-1 polarity ratio. If this value is equal to 1 then the sum of YO-PRO®-1 fluorescence on the “*anode*” side is equal to the “*cathode*” side of the cell. If the value is greater than one then more YO-PRO®-1 fluorescence was present on the “*anode*” side; likewise, if the value is less than one then the “*cathode*” side of the cell exhibits a higher degree of YO-PRO®-1 fluorescence. (**d**) The YO-PRO®-1 polarity ratio plotted over time for UP and BP nsEP exposures. Quantitative statistics- nsEP exposure (N, cells): ↑300 (11,28), ↑600 (11,29), ↑900 (11,28), ↑1,200 (8,27), ↑1,800 (8,28), ↑300↓300 (11,31), ↑600↓600 (11,20), ↑900↓900 (11,31), ↑300↓900 (10,31), and ↑900↓300 (11,31).

Next, we visualized the location of YO-PRO®-1 fluorescence to examine the spatial parameters of membrane breakdown. For these experiments, we placed a line profile over the cell starting at the anode and ending at the cathode side of the cell (Fig. 4a). During a BP nsEP exposure, the anode and cathode are reversed leading to some terminology confusion. For the purposes of this paper, we will refer to the “*anode*” and “*cathode*” side of the cell relative to the first portion of the BP nsEP. From a ↑300 nsEP, we observed that YO-PRO®-1 fluorescence preferentially entered the “*anode*” side, and 20 s post nsEP we observed a spike in YO-PRO®-1 fluorescence on the “*cathode*” side (Fig. 4b). From a symmetrical ↑300↓300 nsEP exposure, we detected a minimal change to YO-PRO®-1 fluorescence that did not present any polarity dependence. From an asymmetrical ↑900↓300 nsEP exposure, we detected an “*anode*” dependence in YO-PRO®-1 fluorescence that after 10 s also exhibited fluorescence on the “*cathode*” side of the cell (Fig. 4b). On the contrary, when the individual pulse durations were reversed with a ↑300 ns front pulse and a ↓900 ns back pulse, we observed distinct YO-PRO®-1 fluorescence on the “*cathode*” side of the cell (Fig. 4b).

To quantify the spatial distribution of YO-PRO®-1 fluorescence, we generated a polarity ratio that divided the sum of YO-PRO®-1 fluorescence from the “*anode*” and “*cathode*” side of the cell at baseline then at 2, 5, 10, 20 and 30 s post nsEP (Fig. 4c). If the ratio equaled one, then this would demonstrate that the distribution of YO-PRO®-1 fluorescence across the cell at any particular time point did not favor either side. Our analysis demonstrated that exposure to symmetric BP nsEPs resulted in unbiased YO-PRO-1 uptake. In contrast, all UP nsEP exposures displayed “*anode*” dependence distinguishable 2 s after the nsEP exposure, and this “*anodal*” bias peaked between 5-10 s post exposure (Fig. 4d). From the asymmetrical BP nsEP exposures, “*anode*” dependence in YO-PRO®-1 fluorescence was present only when the 900 ns pulse duration was assigned to the front pulse (Fig. 4d). Contrarily, when the back pulse was 900 ns, with a front 300 ns pulse, we detected a reversal in maximum YO-PRO®-1 fluorescence to the “*cathode*” (Fig. 4d).

### BPC may be partially mediated by a reduction in nanopore perturbation

**Figure 5:**
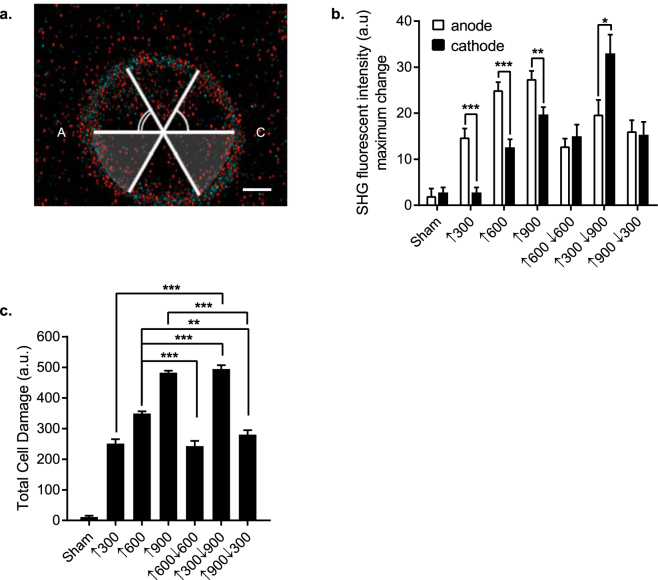
BPC is partially due to a reduction in plasma membrane perturbation. (**a**) Representative image of a CHO-K1 cells utilized for second harmonic generation; scale bar 2.5 µm. (**b**) Quantification of the maximum change in SHG intensity from the “*anode*” and “*cathode*” side of the plasma membrane in physiological solution. Quantitative statistics- nsEP exposure (cells): Sham (10), ↑300 (10), ↑600 (17), ↑900 (14), ↑600↓600 (9), ↑300↓900 (12), and ↑900↓300 (10). Statistical analysis of maximum change in SHG intensity determined with an unpaired two-tailed *t-test*, alpha = 0.05, ***p < 0.0001, **p = 0.0001, and *p = 0.0165. (**c**) Quantification of the total cell damage assessed from SHG imaging of CHO-K1 cells (10 cells exposed per UP or BP nsEP exposure) exposed to UP and BP nsEP in outside physiological. Quantitative statistics- nsEP exposure (cells): Sham (10), ↑300 (11), ↑600 (10), ↑900 (10), ↑600↓600 (10), ↑300↓900 (10), and ↑900↓300 (12). Statistical analysis of maximum change in SHG intensity determined with an unpaired two-tailed *t-test*, alpha = 0.05, ***p < 0.0001, and **p = 0.0001.

Previous work from our lab and others have demonstrated that single harmonic generation (SHG) imaging is capable of measuring rapid and subtle plasma membrane disturbances in living cells, and can be used to directly observe nsEP-induced *nanoporation* ^10,11^. We employed SHG imaging to evaluate the plasma membrane disruption resulting from symmetrical and asymmetrical BP nsEPs. CHO-K1 cells were loaded with Di-4-ANEPPDHQ (Di-4) dye, which embeds itself in the cell membrane, and allows for a uniform SHG signal throughout the cell membrane (Fig. 5a)^10,11^. From a ↑600  nsEP exposure, we averaged this signal across each pole, and observed a 50% difference in the SHG response between the “*anode*” and “*cathode*”, which corroborated previously published data (Fig. 5b)^10^.

We next measured the maximum change in SHG signal from a ↑600↓600 nsEP, and observed an overall reduction in SHG as compared to a ↑600 nsEP (Fig. 5b). We also detected an equivalent loss of SHG signal from the “*anode*” and “*cathode*” side of the cell (Fig. 5b), agreeing with published data^11–13^. From a ↑300↓900 nsEP, we observed a greater loss of SHG signal from the plasma membrane facing the “*cathode*”, and the overall change was larger than from a ↑600 nsEP. A ↑900↓300 nsEP exposure exhibited a largely symmetric SHG loss, and intensity level that was comparable to the ↑600↓600 nsEP (Fig. 5b). When we sum the total attenuation of SHG around the entire cell, termed “Total Cell Damage”, we found that UP nsEP exposures appear to exert more damage to the cell (presumed to be nanoporation) than symmetric BP exposures. This directly supports previous observations using indirect dye uptake methods^7^. Additionally, we observed that more membrane damage is generated when we assign 900 ns to the back pulse width (↑300↓900) as opposed to a ↑900↓300 BP nsEP exposure. This corroborates previous figures were the ↑300↓900 generates more YO-PRO®-1 uptake as opposed to the ↑900↓300 BP nsEP.

### Working model to define how membrane integrity is altered by symmetrical and asymmetrical BP nsEP exposures

**Figure 6:**
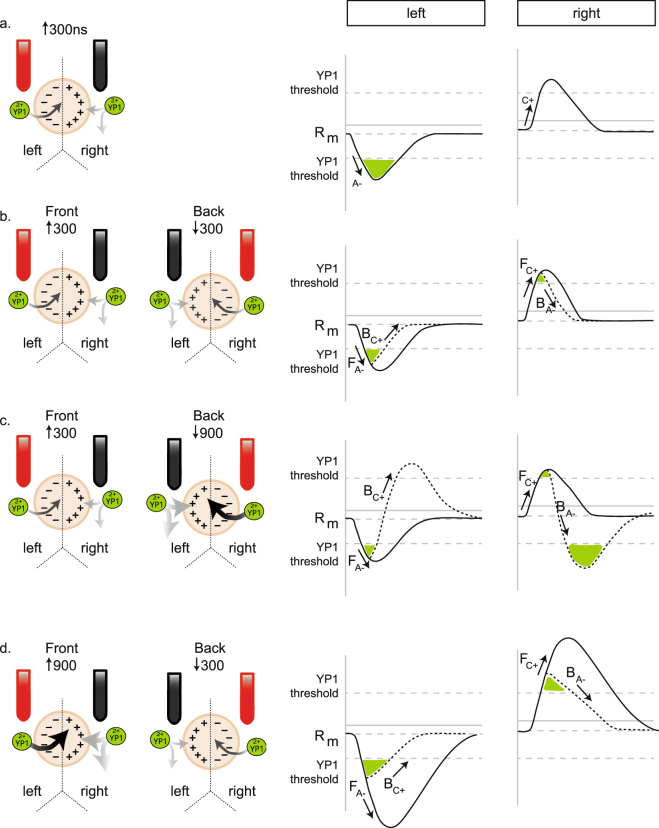
BPC model. (**a**) A schematic of a single cell exposed to a ↑300 UP nsEP. The anode and cathode are placed above the left and right sides of the membrane, respectively. Upon exposure, the left side of the membrane becomes hyperpolarized by the anode (_A−_), and the right side becomes depolarized by the cathode (_C+_). We plot the theoretical changes to the membrane potential under the left and right sides of the cell, respectively. YO-PRO®-1 uptake requires the membrane potential to cross a threshold with the addition of a driving force. As a cationic membrane impermeable dye, YO-PRO®-1 is attracted to the net negative polar end of the cell. From UP nsEP exposure, YO-PRO®-1 exhibits an anodal dependence. (**b**) For a BP nsEP exposure the electrode orientation is opposite on the second pulse. Thereby the BP nsEP consists of a front (F) pulse that generates F_A−_ and F_C+_ on the left and right sides of the cell, respectively. The back (B) pulse consists of a B_A−_, and B_c+_ on the right and left sides of the cell, respectively. For a ↑300↓300 BP nsEP exposure, the additional B_A−_ and B_c+_ components will generate an equivalent effect as the front pulse, but opposite in orientation. The B_A−_ will counteract F_C+_, and expedite the membrane potential to return to baseline. The B_c+_ counteracts F_A−_, which circumvents YO-PRO®-1 uptake. (**c**) Under a ↑300↓900 nsEP exposure, the left side of the membrane exhibits a driving force for YO-PRO®-1 uptake due to the front ↑300 ns pulse. The right side of the membrane exhibits a stronger driving force due to the back ↓900 ns pulse. Our theoretical changes to local membrane potential suggest that F_C+_ increases the membrane potential but the hyperpolarization initiated by B_A−_ causes YO-PRO®-1 uptake on the right side of the cell. (**d**) For a ↑900↓300 BP nsEP exposure, F_A−_ induced by the ↑900 ns pulse causes the membrane potential to move past the YO-PRO®-1 uptake threshold, but is limited by the ↓300 ns B_C+_ that circumvents overall “*anodal*” charging. Along the right side, the ↑900 ns pulse F_C+_ depolarizes the membrane, and the ↓300 ns B_A−_ advances its return to a resting baseline.

In previous sections, we presented how symmetrical BP nsEP exposures can generate an attenuated physiological response compared to a UP pulse. We also demonstrated that a ↑900↓300 asymmetric BP nsEP exposure generated *BPC*; whereas the opposite configuration (↑300↓900) does not. To explain these findings, we designed a conceptual model, expanding on a previous concept presented by Pakhomov *et al*.^7^ Our schematic encompasses the orientation of the bipolar electrode that delivers the nsEP, and the polarity shift it induces within the cell. In Fig. 6a, we demonstrate that a UP nsEP hyperpolarizes and depolarizes the cell membrane under the anode and cathode electrode, respectively. In this scenario, the charging effects initiated by a UP nsEP exposure reach their maximum changes to membrane potential then return to baseline overtime. We describe the hyperpolarization due to the anode as _A−_, and depolarization by the cathode as _C+_. If _A−_ and _C+_ are sufficient it will drive membrane potential past a threshold to allow YO-PRO®-1 uptake, but its entry is determined by the presence of a driving force. As an impermeable cation, YO-PRO®-1 entry is driven by _A−_, because it generates a negatively charged local membrane allowing the dye to move down its electrochemical gradient. As a result, YO-PRO®-1 uptake exhibits an anodal dependence from a UP nsEP exposure as similarly observed with other cationic uptake dyes (i.e. Propidium iodide)^6–8,14^. The overall extent of YO-PRO®-1 uptake is regulated by the magnitude of _A−_, which in turn is effected by nsEP parameters such as applied voltage, pulse duration, and its delivery as a UP or BP waveform. In BP nsEP exposure, the electrophysiological effects initiated by the front pulse are similar to a UP presentation, but restricted by the back nsEP.

In our model, a BP nsEP consists of a front (F) and back (B) pulse. The front pulse initiates F_A−_ and F_C+_ on the left and right sides of the cell, respectively (Fig. 6b). The delivery of the back pulse is different, because the electrode orientation is switched causing B_A−_ and B_C+_ to occur on the right and left sides of the cell, respectively. The charging reversal by the back pulse counteracts the front pulse charging effects on the cell membrane. These overlapping changes to the membrane potential may explain the *BPC* effect driven by symmetrical BP nsEP exposures, and how an asymmetrical BP nsEP exposure can modify it (Fig. 6b). We graphically illustrate this concept for a symmetrical ↑300↓300 nsEP exposure (Fig. 6b). From the front ↑300 ns pulse, F_A−_ and F_C+_ effect the left and right sides of the cellular membrane, respectively; however, the back ↓300 ns pulse applies B_A−_ and B_C+_ to the right and left sides of the cell, respectively (Fig. 6b). As a result, the charging effects driven by the front pulse are attenuated by the back pulse thus minimizing the overall time the membrane potential is past the YO-PRO®-1 uptake threshold. Furthermore, B_A−_ on the right side of the cell causes a driving force for YO-PRO®-1 uptake (Fig. 6b). This results in dye uptake on both sides of the cell, but the extent of YO-PRO®-1 uptake will be attenuated due to the charging reversal presented by the back pulse. The idea that the reversed placement of B_A−_ and B_C+_ can attenuate the front pulse charging components yields a potential mechanism for *BPC*. Furthermore, it highlights key variables to explain how *BPC* maybe altered by individually modifying the front and back nsEP parameters.

For a ↑300↓900 nsEP exposure, our working model suggests that YO-PRO®-1 uptake driven by the ↓900 ns back pulse yields a significant impact on the membrane (Fig. 6c). In this scenario, the front ↑300 ns pulse yields F_A−_ and F_C+_ on the left and right sides of the cell, respectively. However, B_A−_ and B_C+_ significantly counters these initial changes, because it is driven by a↓900 ns pulse nsEP that is three-fold longer in duration. As a result, the left side of the cell is marginally hyperpolarized by the ↑300 ns pulse, and significantly depolarized by the ↓900 ns pulse to reverse the membrane potential past baseline, and over the YO-PRO®-1 uptake threshold (Fig. 6c). On the right side of our model cell, the membrane is initially depolarized by the front pulse, but immediately hyperpolarized past baseline, and over the YO-PRO®-1 uptake threshold (Fig. 6c). On both sides of the cell, the membrane potential has crossed the hyperpolarization and depolarization YO-PRO®-1 thresholds, but a driving force is only present from _A−_ (Fig. 6c). In this scenario, dye uptake on the left side of the cell that is generated by F_A−_ is negatively affected by B_C+_, thereby *cancelling* the maximal response. On the right side, B_A−_ generated by the ↓900 pulse reverses the membrane potential due to F_C+_, and allows significant YO-PRO®-1 uptake. On the contrary, when the front pulse become the ↑900 ns duration the overall effect on YO-PRO®-1 is different (Fig. 6d). The front ↑900 ns pulse yields significant F_A−_ and F_C+_ on the left and right sides of the cell, respectively. However, the ↓300 ns pulse exposure *cancels* the maximal YO-PRO®-1 uptake due to the front pulse by reversing the membrane potential *via* B_A−_ and B_C+_ on the right and left sides of the cell, respectively (Fig. 6d).

## Discussion

Long duration nsEP widths were previously utilized to demonstrate that Ca^2+^ influx initialed by a ↑300 nsEP can be *cancelled* by the delivery of a ↑300↓300 nsEP waveform^7^. In this report, we demonstrated that the negative impact on membrane integrity triggered by a ↑300 nsEP exposure can be *cancelled* by a ↑300↓300 nsEP. In addition, our observations provide further insight demonstrating that CHO-K1 cells exposed to ↑600↓600 and ↑900↓900 nsEP also exhibited reduced YO-PRO®-1 uptake compared to a ↑600 and ↑900 nsEP exposure, respectively. While the delivery of these BP nsEPs reduced YO-PRO®-1 uptake, the values did increase with the BP pulse duration suggesting *BPC* may be a secondary effect following initial YO-PRO®-1 uptake from the front pulse. Together these results demonstrate that *BPC* is not isolated to a particular nsEP width, but exists across various pulse durations in the nanosecond regime.

Our data addressed if symmetrical pulse widths are required for a BP nsEP to *cancel* the physiological response of a UP nsEP. From the ↑300↓900 nsEP exposure, we detected no significant reduction in YO-PRO®-1 uptake compared to the ↑600 nsEP. On the contrary, the ↑900↓300 nsEP generated a significant reduction in YO-PRO®-1 uptake. What drives *BPC* in the ↑900↓300 but not the ↑300↓900 nsEP exposure despite both exhibiting a doubling of total pulse duration? Previous models propose that a symmetrical BP nsEP exposure causes entry of small molecules on one end of the cell, and drives molecules out on the other side, suggesting *cancellation* is due to an equivalent gain and loss of small ions^7^. When we applied the same rules to a ↑300↓900 asymmetrical nsEP exposure we predicted that uptake of ions from the front ↑300 nsEP would be coupled with a *push* from the ↓900 nsEP leaving a residual of 600 ns. In fact, the YO-PRO®-1 uptake generated from the ↑300↓900 BP nsEP is not significantly different from the UP ↑600 ns exposure validating the theory behind the model^7^. If we apply the same logic for the ↑900↓300 exposure we would expect the same finding, but interestingly YO-PRO®-1 uptake is significantly reduced from this exposure. One potential factor that may explain the differential physiological responses may be the role of the front pulse duration. An alternative hypothesis is that a subcellular process becomes saturated by the ↑900 nsEP, but exposure to the ↑300 nsEP allows further subcellular cascade activation potentiating YO-PRO®-1 uptake. Additionally, while some attention has been paid to the frequency components of BP versus UP pulses, this large difference between the effects driven by the ↑300↓900 and ↑900↓300 BP nsEP exposures would appear to reduce the likelihood that frequency components within each pulse are playing a dominant role^6,12,13,15^.

A residual parameter of symmetrical *BPC* on a UP nsEP (i.e. ↑600 vs. ↑600↓600 nsEP) is that the BP nsEP has a total pulse duration twice the UP nsEP. Our data evaluated this parameter, and determined *BPC* still occurs with the delivery of a back pulse that is a third of the front pulse width, and comprises just 25% of the total BP pulse duration (i.e. ↑900↓300 nsEP vs ↑900 nsEP). On the other hand, the delivery of a back nsEP that is three times longer than the front pulse width, encompassing 75% of the total BP nsEP pulse duration (i.e. ↑300 ns↓900 ns BP nsEP vs. ↑300 nsEP) generated a significant increase in YO-PRO®-1 uptake compared to the ↑300 nsEP. What is the driving mechanism(s) between these two observations? Thus far, we only detected *BPC* when the individual BP nsEP widths are symmetrical or the front pulse is orders of magnitude larger than the back pulse. Perhaps the mechanism behind *BPC* is differentially affected by the energy deposited through the front and back pulses. Previous work using asymmetrical bipolar microsecond pulse durations demonstrated that asymmetric BP pulses generate more efficient uptake of small molecules^8,9^. Conceivably the placement of the ↓900 ns second pulse is unaffected by *BPC*, and as a result causes additional uptake of YO-PRO®-1 on localized membrane otherwise not observed by delivery of a UP nsEP.

Our spatiotemporal analysis revealed localized YO-PRO®-1 fluorescence varied across the different asymmetrical BP nsEP exposures. Delivery of the ↑900↓300 nsEP exposure generated an “*anodal*” dependence similar to UP nsEP delivery; however, the ↑300↓900 nsEP exposure generated an opposite spatial dominant YO-PRO®-1 fluorescence. We detected these differences from two quantitatively distinct approaches by 1) mapping the peak YO-PRO®-1 fluorescence values across the cell, and 2) summation of “*cathode*” and “*anode*” fluorescent values to form a polarity ratio. The spatial maps between the ↑300↓900 and ↑900↓300 nsEP exposure are the first lines of evidence to suggest that despite these equivalent total pulse durations the individual pulse orders generate unique localized YO-PRO®-1 fluorescence across the cell. When we cross verified these spatial results with the extent of membrane damage reported by SHG imaging, we observed that the results compare well. Specifically, peak membrane damage (via SHG) and maximum YO-PRO®-1 uptake generated from the ↑900↓300 nsEP exposure were present on the same “*anode*” side of the cell as the ↑900 nsEP exposure. While the ↑300↓900 nsEP generated these peak metrics on the opposite “*cathode*” side of the cell. This divergent spatial property of YO-PRO®-1 fluorescence maybe the reason why we see a loss of *BPC* from the ↑300↓900 nsEP exposure compared to ↑900↓300 nsEP.

Our working model suggests *BPC* is generated by the reversed placement of B_A−_ and B_C+_. In our schematic, a nsEP exposure can depolarize or hyperpolarize the membrane potential past a threshold for YO-PRO®-1 uptake. At this threshold, channel activation, nanopores, or both lead to a membrane perturbation potentially allowing the impermeable YO-PRO®-1 dye entry into the cell. The final variable for dye uptake is a driving force. We suggest that YO-PRO®-1 uptake is driven by an increase of negative charge within the cell relative to the outside environment. Unlike membrane dyes (i.e. FluroVolt) that likely respond to the shift in ionic flux to establish membrane potential. YO-PRO®-1 uptake is very slow as maximum dye uptake isn’t reached until 30 s + after nsEP exposure^16^. From our schematic, we suggest that YO-PRO®-1 uptake due to hyperpolarization may bias a trend for more dye uptake despite a comparable change due to depolarization. This would likely be due to the slightly more negative resting membrane potential, but future studies are required to test for a significant contribution. A working hypothesis generated from this model is that increasing the interpulse interval between the BP nsEPs will delay delivery of back pulse charge components on the “*anode*” and “*cathode*” sides of the cell resulting in the allowance of more dye uptake. Previous work with a symmetrical ↑300↓300 nsEP exposure demonstrated Ca^2+^ and YO-PRO®-1 uptake can be increased by lengthening the interpulse intervals, which maybe also true for the asymmetrical BP nsEP^7,17^. While our model utilizes the charging, and discharging effects generated by nsEP exposure as the cause of small ion uptake, one possibility is that this action may be due to nanopore formation. Thereby an outstanding question is what component of nsEP exposure is the key player to *BPC*- nanopore formation or membrane charging/discharging? To address this question will require an understanding of the electrical and biophysical components generated by nsEP exposure, and identifying their specific bioeffects on the cell^3,18,19^.

## Materials and Methods

### Cell line and propagation

Chinese hamster ovarian-K1 (CHO-K1) cells (#CCL-61, American Type Culture Collection, Manassas, VA) were seeded in a T75 flask maintained at 37 °C with 5% CO_2_ in F12K (D8437, Sigma-Aldrich) media containing 10% fetal bovine serum (FBS), 100 IU/mL penicillin and 0.1 µg/mL streptomycin. To prepare CHO-K1 cells for nsEP exposures, the cells were passaged upon 70-80% confluency. Our sub-culturing procedure consisted of two washes in Dulbecco’s Phosphate Buffer Solution (DPBS) without magnesium chloride (MgCl_2_) and calcium chloride (CaCl_2_), followed by 5mLs of 0.05% Trypsin to release the adherent cells from the flask. The trypsination process was terminated with the addition of 5 mLs of CHO-K1 media, and the cell suspension was transferred to a 15 mL conical tube. A 10 µL aliquot of the cell suspension sample was combined with 10 µL of Trypan Blue to exclude dead cells from the cell density calculation. For experiments, 30,000 cells were plated on a 35 mm dish with a 10 mm glass bottom insert pre-coated with poly-d-lysine (Mat-Tek Corporation, P35GC-0-10-C). After 24-48 hours, the cells were exposed to nsEP for live-imaging experiments.

### Nanosecond Electric Pulse Exposure and Confocal Microscopy

A nsEP was delivered to a custom microelectrode composed of two parallel 100 µm diameter tungsten electrodes separated by 150 µm and positioned with a micromanipulator (MPC-200, Sutter Inc. Novato California), 50 µm above the CHO-K1 cells at a 45° angle^3,20^. The nsEP generator has been previously described^6,21^. In short, the custom nsEP pulse system consisted of a capacitor discharge circuit driven by a high voltage power supply generating UP and BP nsEPs at an amplitude of 350 Volts (V) that generated an electric field of 12.0 kV/cm. A Stanford DG535 digital pulse generator (Stanford Research Systems, Sunnyvale, California) was used to adjust the UP nsEP width, and the individual front and back pulses of the BP pulse. The pulse generator was programmed to trigger a LSM-710 Zeiss confocal microscope (Carl Zeiss MicroImaging GmbH, Germany) to initiate imaging, and after a preset delay, signaled a HP8112A pulse generator to deliver a single pulse exposure to the cells. To verify delivery of the nsEP (voltage amplitude and pulse width), we monitored the waveform on a Tektronix TDS3052 500-MHz oscilloscope (Tektronix Inc, Beaverton, Oregon) for each exposure. For all experiments, confocal micrographs were captured at 512pixels^2^ (1 frame/ second) for 40 seconds (s) (10 s baseline + 30 s post-nsEP) with a 40x oil-immersion objective.

Second harmonic generation imaging was completed with resonant scanning through a scan head of a modified model No. TCS SP5 II (Leica Geosystems, Norcross, GA), which was coupled to a Ti:Sapphire oscillator at 980 nm (Coherent Chameleon, 130fs, 80 MHz, ~15 mW at the sample; Coherent Laser, Santa Clara, CA). The Second Harmonic Generation (SHG) image was collected in transmission mode by a photomultiplier tube after passing through 680 nm short pass and 485/25 nm bandpass filters. A more detailed description of this imaging system is presented in previous publications^10,11^.

### YO-PRO®-1 fluorescent staining

CHO-K1 cells plated on 35 mm glass-bottom Mat-Tek dishes were prepared for fluorescent imaging with the following procedure. To begin, we syphoned off the CHO-K1 cell media, and rinsed the cells three times in outside physiological solution (2 mM MgCl_2_, 5 mM KCl, 10 mM HEPES, 10 mM Glucose, 2 mM CaCl_2_, and 135 mM NaCl; pH of 7.4 and 290 mOsm) before adding 1 mL of outside physiological solution containing 2µLs (5 µM) of YO-PRO®-1 (Y3603, Fisher Scientific). The cells were incubated in YO-PRO®-1 containing outside physiological solution for ten minutes followed by 30 minutes of confocal imaging combined with nsEP exposures; at room temperature. All UP and BP nsEP parameters were randomly selected, and each nsEP exposure was separated by 700-1,000 µm across the 10 mm glass coverslip.

### Di-4-ANEPPDHQ loading for SHG Imaging

CHO-K1 cells were placed in 35 mm poly-l-lysine-coated, glass-bottomed dishes (MatTek, Ashland, MA), and incubated for 30 minutes in growth media to allow adherence. Before loading, the cells were rinsed with outside physiological solution. For imaging, 5 µM final concentration Di-4-ANEPPDHQ (Life Technologies, Grand Island, NY) was added and incubated for an additional 30 minutes. The experiment was performed in the labeling solution to limit any diffusion of the probe molecules out of the cell membrane.

### Image Processing and Analysis

All confocal micrographs were uploaded into ImageJ image analysis software as a hyperstack containing 40 images^22^. From the DIC images, cells were outlined and archived with an Image J region of interest (ROI) tool manager. The fluorescent data from each of the 40 images were collected in series per cell across each nsEP exposure parameter. The raw data was exported to an Excel file and copied into GraphPad Prism 6.00 for Macintosh (GraphPad Software, La Jolla California USA). In GraphPad Prism, YO-PRO®-1 uptake from each cell was calculated by subtracting the average baseline YO-PRO®-1 fluorescence value from each pre- and post- exposure data point. As a result, YO-PRO®-1 uptake values at baseline centered around zero reflecting its entry only after nsEP exposure (Equation 1). All statistical tests were completed using GraphPad Prism, and all data was plotted as mean ± standard error of the mean (SEM). CHO-K1 cells were exposed to a combination of 25 different UP and BP nsEP parameters. Statistical analysis consisted of an unpaired two-tailed *t-test*, alpha = 0.05.

1 $$\mathrm{YO} \mbox{-} \mathrm{PRO}{\rm{\circledR }} \mbox{-} 1\,{\rm{uptake}}={\rm{value}}-{\rm{average}}({\rm{baseline}})$$

### Equation 1. YO-PRO®-1 uptake

YO-PRO®-1 fluorescence values were calculated every second (s) during a 10s-baseline followed by 30s after the nsEP exposure. The baseline fluorescence signals were averaged, and this number was subtracted from each data point collected during the experiment to derive a YO-PRO®-1 uptake value. Consequently, YO-PRO®-1 uptake is approximately zero at baseline, and denotes the addition of YO-PRO®-1 dye that entered the cell after nsEP exposure.

### Spatiotemporal measurements of YO-PRO®-1 uptake

To measure the spatial parameters of YO-PRO®-1 fluorescence we used the Line tool in Image J and placed a line across the middle portion of the cell beginning at the “*anode*” and ending at the “*cathode*” side of the cell. The raw fluorescent values were archived into an Excel spreadsheet, and processed in GraphPad Prism 6.0 for Macintosh where line profiles were filtered in GraphPad Prism’s smooth line analysis; smooth value = 4 for illustration. The YO-PRO®-1 uptake polarity ratio was calculated by dividing the fluorescence values of the line profile evenly into two groups then collecting the sum from these two groups. The “*cathode*” and “*anode*” summations were then divided by each other to generate a polarity ratio. This process was completed at baseline then at 2, 5, 10, 20, and 30 s post nsEP exposure to generate the YO-PRO®-1 polarity ratio over time.
